# Transcriptome Analysis Reveals Metabolic Pathways and Key Genes Involved in Oleic Acid Formation of Sunflower (*Helianthus annuus* L.)

**DOI:** 10.3390/ijms26146757

**Published:** 2025-07-15

**Authors:** Yingnan Mu, Ying Sun, Yang Wu, Liuxi Yi, Haifeng Yu, Shaoying Zhang

**Affiliations:** 1 Agricultural College, Inner Mongolia Agricultural University, Hohhot 010019, China; muyingnan19900222@163.com (Y.M.); 2678048214@imau.edu.cn (Y.S.); wuyang@imau.edu.cn (Y.W.); yiliuxivip@163.com (L.Y.); 2 Inner Mongolia Academy of Agricultural & Animal Husbandry Sciences, Hohhot 010031, China

**Keywords:** fatty acids, molecular mechanism, qRT-PCR, RNA-Seq, sunflower

## Abstract

Sunflower is one of the four most important oilseed crops in the world, and its edible oil is of high nutritional quality. However, the molecular regulatory mechanism of oil accumulation in sunflowers is still unclear. In this study, we selected two inbred lines with significant differences in oleic acid content and similar agronomic traits: the high oleic acid content (82.5%) inbred line 227 and the low oleic acid content (30.8%) inbred line 228. Sunflower seeds were selected for transcriptome experiments at 10, 20, and 30 days after full bloom (DAFB). There were 21, 225, and 632 differentially expressed genes (DEGs) identified at the three times, respectively. The Gene Ontology (GO) analysis showed that DEGs from two sunflower cultivars at three stages were significantly enriched in the activities of omega-6 fatty acid desaturase and glucosyltransferase. Kyoto Encyclopedia of Genes and Genomes (KEGG) analysis found that at 10, 20, and 30 DAFB, DEGs were significantly enriched in the unsaturated fatty acid synthesis pathway, glutathione metabolism pathway, and pyruvate metabolism pathway. Through mapping analysis of GO in the KEGG pathway, it was found that the omega-6 fatty acid desaturase gene *FAD6/FAD2*, diacylglyceroyltransferase gene *DGAT*, glycerol-3-phosphate acyltransferase gene *GPAT*, and long-chain acyl-CoA synthase gene *LACS* may play important roles in regulating sunflower oleic acid content. Our research provides candidate genes and a research basis for breeding high oleic sunflowers.

## 1. Introduction

Sunflower (*Helianthus annuus* L.), native to southwestern North America, is an annual herbaceous plant that belongs to the Asteraceae family. It is an important oil crop and ornamental plant, as well as an important bioenergy material. Sunflower seed oil is a high-quality vegetable oil used in daily consumption and food processing. Sunflowers metabolize and synthesize various fatty acids (FAs), which are mainly composed of oleic acid (C18:1), linoleic acid (C18:2), palmitic acid (C16:0), and stearic acid (C18:0) [1]. Sunflower seed has a relatively low content of saturated fatty acids (palmitic acid and stearic acid), ranging from 4% to 13%, and a high content of unsaturated fatty acids (oleic acid and linoleic acid), ranging from 87% to 94% [2,3]. The content of oleic acid and linoleic acid is usually negatively correlated. As early as 1999, the Food and Agriculture Organization of the United Nations classified sunflowers into three major genotypes based on their oleic acid content: low oleic sunflowers (traditional sunflowers) with oleic acid content ranging from 14% to 39%; Sunflowers with medium oleic acid content, with oleic acid content ranging from 42% to 72%; Sunflowers with high oleic acid content, with oleic acid content ranging from 75% to 91%. The sunflower oil raw materials on the market now mainly come from traditional sunflowers, with linoleic acid accounting for 55%~65% of unsaturated fatty acids and oleic acid accounting for 20%~30%.

**Figure 1:**
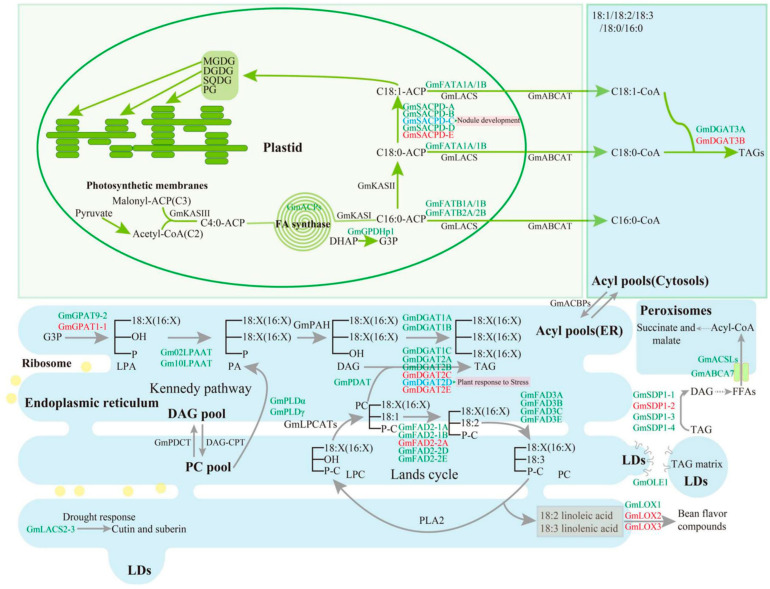
Fatty acid biosynthesis, triacylglycerol assembly, and degradation pathways [14]. The yellow dots and green squares, in the figure, represent the ribosome and transmembrane proteins (ATP-binding c assette (ABC) transporter, GmABCA7), respectively. Abbreviations for substrates: DAG, diacylglycerol; DGDG, digalactosyldiacylglycerol; DHAP, dihydroxyacetonephosphate; FFAs, free fatty acids; G3P, glycerol-3-phosphate; LPA, lysophosphatidic acid; LPC, lysophosphatidylcholine; MGDG, monogalactosyldiacylglycerol; PA, phosphatidic acid; PC, phosphatidylcholine; PG, phosphatidylglycerol; SQDG, sulfoquinovosyldiacylglycerol; TAG, triacylglycerol. Abbreviation for proteins: ABCA or ABCAT, ATP-binding cassette (ABC) A transporter; ACBPs, acyl-CoA-binding proteins; ACP, acyl carrier protein; ACSLs, long-chain acyl-CoA synthetase; CPT, CDP-choline:diacylglycerol cholinephosphotransferase; DGAT, acyl-CoA:diacylglycerol acyltransferase; FAD2, omega-6-desaturase 2; FAD3,omega-3desaturase 3; FATA, acyl-ACP thioesterase A; FATB, acyl-ACP thioesterase B; GPAT, glycerol-3-phosphate acyltransferase; GPDHp1, glycerol-3-phosphate dehydrogenase 1; KAS, 3-ketoacyl-[acyl carrier protein] synthase; LACs, long-chain acyl-CoA synthetase; LOX, lipoxygenase; LPAAT, lysophosphatidic acid acyltransferase; LPCAT, lysophosphatidylcholine acyltransferase; OLE1, oleosin 1; PAP (or PAH), phosphatidic acid phosphatase; PDAT, phospholipid:diacylglycerol acyltransferase; PDCT, phosphatidylcholine:diacylglycerol cholinephosphotransferase; PLA, phospholipase A; PLD, phospholipase D; SACPD, stearoyl-ACP desaturase; and SDP1, SUGAR-DEPENDENT1.

The composition of fatty acids largely depends on lipid metabolism pathways and their regulatory networks [4,5]. Fatty acids are synthesized from acetyl-CoA in plastids, then exported to the cytoplasm, and finally synthesized in the endoplasmic reticulum (ER) (Figure 1) [6]. After the removal of acyl carrier protein (ACP) using acyl-ACP thioesterase, fatty acids are activated by long-chain acyl-CoA synthase (LACS), which are then transported to the endoplasmic reticulum (ER) to assemble into glycerol lipids. The generated 18:1 CoA is transferred to phosphatidylcholine (PC), where it is further desaturated by fatty acid desaturases (FADs) FAD2 and FAD3 in the ER to linoleic acid and linolenic acid (Figure 1) [7]. A high proportion of unsaturated fatty acids in the human diet is beneficial for cardiovascular health. The proportion of oleic acid and linoleic acid in sunflower seed oil is the key factor determining its quality. Currently, research on the molecular regulation mechanism of sunflower oil metabolism is not deep enough and needs further investigation.

Transcriptomic analysis is a revolutionary and powerful tool that enables researchers to identify genes and pathways related to plant resistance to stress and growth and development [8,9,10]. Transcriptome analysis of *Arundo Donax* L. under salt stress conditions revealed that hormone regulation, reactive oxygen species (ROS) clearance, and osmotic fluid biosynthesis play important roles in salt stress response [8]. Transcriptome analysis of cotton leaves inoculated with biocontrol agent *Fusarium oxysporum* 082 at 12, 24, and 48 h revealed that *F*. *oxysporum* 082 can stimulate ROS and MAPK to participate in defense responses. It was also found that the genes encoding ERF, FLS2, MYB, GST, and CML play important roles in resisting Verticillium wilt [11]. Transcriptome analysis of *Arabidopsis thaliana* overexpressing the oxalate decarboxylase gene *Odx_S12* revealed that this gene enhances resistance to Verticillium wilt by inducing reactive oxygen species bursts and salicylic acid-induced defense responses [12]. Transcriptome analysis of rapeseed under low temperature stress revealed that flavonoid biosynthesis pathway genes *DFR*, *ANS*, *F3H*, *FLS1*, *CHS1*, *CHS3*, and *TT8*, as well as defense mechanism-related genes *DFR*, *SNL6*, and *TKPR1*, play a role in response to low temperature stress [13].

**Figure 2:**
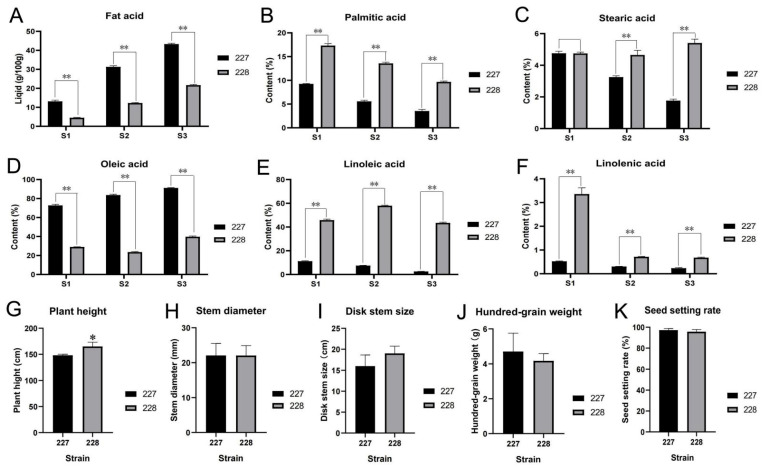
The relative content of major fatty acids in the 227 and 228 strains. (**A**) Fat acid, (**B**) Palmitic acid, (**C**) Stearic acad, (**D**) Oleic acid, (**E**) Linoleic acid, (**F**) Linolenic acid, (**G**) Plant height, (**H**) Stem dlameter, (**I**) Disk stem size, (**J**) Hundred-grain weight, (**K**) Seed setting rate. ** indicates extremely significant level, * indicates significant level.

This study focuses on high oleic acid (82.5%) material 227 and low oleic acid (30.8%) material 228 as research objects (Figure 2). Samples were collected at 10, 20, and 30 DAFB, and the high-throughput sequencing technology (RNA-seq) was used to analyze the gene expression of the two materials. By comparing and analyzing the differential expression of genes, we aim to discover the key functional genes that regulate the oleic acid content in sunflowers and further reveal the molecular mechanism and regulatory rules of sunflower oleic acid formation.

## 2. Results

### 2.1. Analysis of Fatty Acid Content

The phenotype and relative content of major fatty acids (oleic acid, linoleic acid, stearic acid, linolenic acid, and palmitic acid) in the 227 and 228 inbred lines were determined (Figure 2). Although the plant height of the 228 inbred line is significantly higher than that of the 227 inbred line (Figure 2G), there is no significant difference between the 227 and 228 inbred lines in stem thickness, disk stem size, hundred grain weight, and seed setting rate (Figure 2H–K). At 10, 20 and 30 DAFB, the fatty acid content of the 227 strain was 13.1, 31.3 (g/100 g), and 43.3 (g/100 g), respectively, while the fatty acid content of the 228 strain was 4.5 (g/100 g), 12.3 (g/100 g), and 21.7 (g/100 g) (Figure 2A). Among them, the oleic acid content of the 227 strain is 72.8%, 83.7%, and 91.2%, while the oleic acid content of the 228 strain is 29.0%, 23.6%, and 39.8%, respectively (Figure 2D). The linoleic acid content of the 227 strain is 11.2%, 7.5%, and 2.6%, while the linoleic acid content of the 228 strain is 45.8%, 58.0%, and 43.5%, respectively (Figure 2E). The palmitic acid content of the 227 strain is 9.3%, 5.6%, and 3.5%, while the palmitic acid content of the 228 strain is 17.3%, 13.6%, and 9.7%, respectively (Figure 2B). The stearic acid content of the 227 strain is 4.8%, 3.3%, and 1.8%, while the stearic acid content of the 228 strain is 4.7%, 4.7%, and 5.4%, respectively (Figure 2C). The linolenic acid content of the 227 strain is 0.5%, 0.3%, and 0.2%, while the linolenic acid content of the 228 strain is 3.4%, 0.7%, and 0.7%, respectively (Figure 2F). The results showed that the palmitic acid, linoleic acid, and linolenic acid content of the 227 strain was extremely significantly lower than that of the 228 strain, and the oleic acid content of the 227 strain was extremely significantly higher than that of the 228 strain (Figure 2).

### 2.2. Sequencing Quantity, Quality, and New Transcripts

Transcriptome sequencing was performed on sunflower seed samples (18 samples, 3 replicates) from three different stages (10, 20, and 30 DAFB) of 227 inbred line and 228 inbred line. The minimum correlation between the three replicates was 95.5% (Table S2). Each sample produced an average of 6.29 Gb of data, with a minimum Q20 value of 97.41% and a minimum Q30 value of 92.98% for clean reads. The average GC content is 46.33%, and there is no obvious separation phenomenon (Table S2). The results indicate that the sequencing quality meets expectations and can be used for subsequent analysis.

### 2.3. RNA-Seq Sequencing Repeatability Test

**Figure 3:**
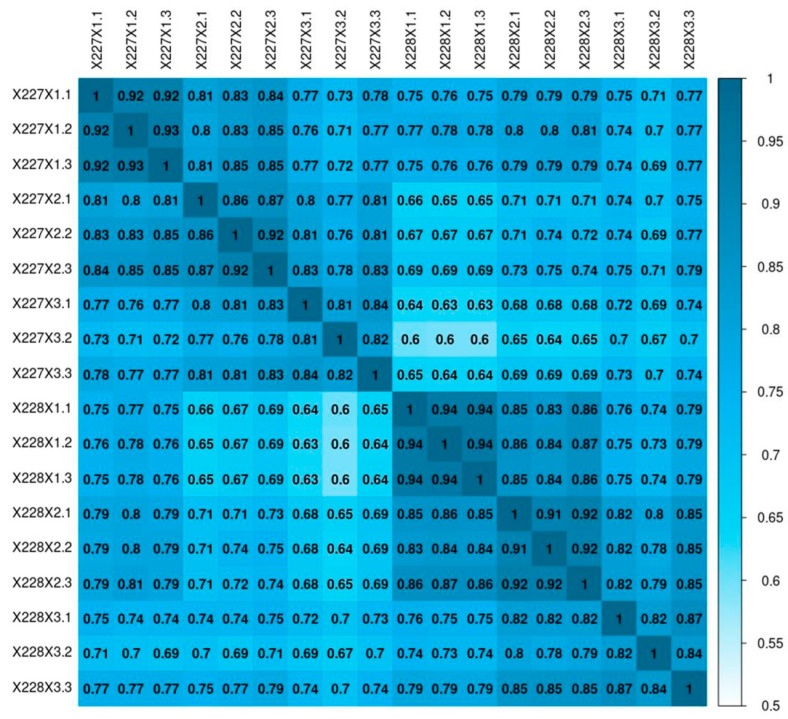
The analysis of sample correlation. Heat map analysis of sample correlation. X2271.1, X2271.2 and X2271.3 stand for the samples of the 227 inbred line at 10 DAFB; X2272.1, X2272.2 and X2272.3 stand for samples of the 227 inbred line at 20 DAFB; X2273.1, X2273.2 and X2273.3 stand for the samples of the 227 inbred line at 10 DAFB; X2281.1, X2281.2 and X2281.3 stand for the samples of the 228 inbred line at 10 DAFB; X2282.1, X2282.2 and X2282.3 stand for the samples of the 228 inbred line at 20 DAFB; X2283.1, X2283.2 and X2283.3 stand for samples of the 228 inbred line at 30 DAFB.

Based on the gene expression levels of each sample, the square of the Pearson correlation coefficient (R^2^) was calculated to determine the correlation between duplicate samples. The results showed that the correlation coefficients between biological replicates of the samples ranged from 0.81 to 0.94 (Figure 3). The correlation coefficients were all higher than 0.8, indicating good reproducibility of the samples in this study.

**Figure 4:**
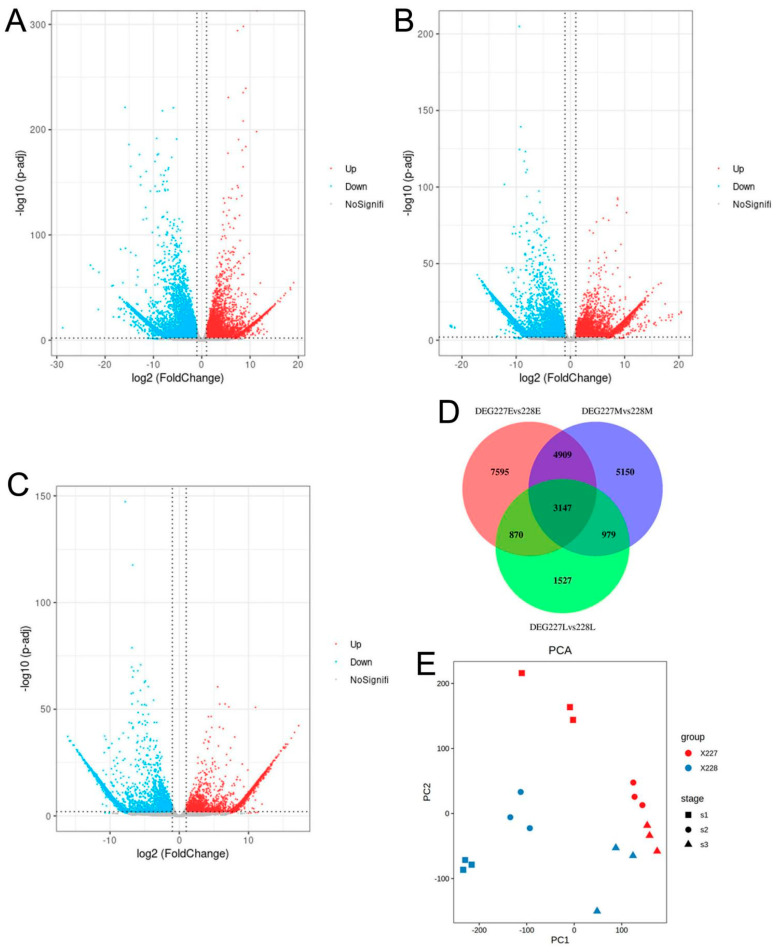
The differences in gene expression between the 227 and 228 inbred lines at 10, 20, and 30 DAFB. (**A**) The volcanic diagram of DEGs between the 227 and 228 inbred lines at 10 DAFB; (**B**) The volcanic diagram of DEGs between the 227 and 228 inbred lines at 20 DAFB; (**C**) The volcano plot of DEGs between the 227 and 228 inbred lines at 30 DAFB; (**D**) The venn diagram of gene expression differences between the 227 and 228 inbred lines at 10, 20 and 30 DAFB, respectively. DEG227Evs228E stands for the DEGs between the 227 and 228 inbred lines at 10 DAFB; DEG227Mvs228M stands for the DEGs between the 227 and 228 inbred lines at 20 DAFB; DEG227Lvs228L stands for the DEGs between the 227 and 228 inbred lines at 30 DAFB. (**E**) The principal component analysis of the samples of 227 and 228 inbred lines at 10, 20, and 30 DAFB. S1, S2, and S3 stand for the stages of 10, 20, and 30 DAFB.

The results of PCA analysis showed that three replicates of the same sample under the same time were clustered together, and different samples and different periods of time were farther apart, indicating the reliability of gene expression analysis (Figure 4E).

### 2.4. Gene Expression Level Analysis

According to the FPKM value of sample genes, the genes were classified into three types: high expression genes (FPKM ≥ 10), medium expression genes (1 < FPKM < 10), and low expression genes (FPKM ≤ 1). The results showed that between the 227 and 228 inbred lines, the gene expression levels at 10 DAFB were higher than those at 20 and 30 DAFB. The gene expression levels of the 228 inbred line showed a trend higher than that of the 227 inbred line among the 10, 20, and 30 DAFB (Table S3 and Figure S1).

### 2.5. Detection and Cluster Analysis of Differential Genes

The DEGs were obtained by screening the gene expression levels according to a threshold. Based on the FPKM values, the expression of all genes at 10, 20, and 30 DAFB of the 227 and 228 inbred lines was compared. The results showed that there were 16,521 DEGs at 10 DAFB (Figure 4A,D), with 9030 genes that were downregulated and 7491 genes that were upregulated. There were 14,185 DEGs at 20 DAFB (Figure 4B,D), with 6595 genes that were downregulated and 7590 genes that were upregulated. There were 6523 DEGs at 30 DAFB, of which 3177 genes were downregulated and 3346 genes were upregulated (Figure 4C,D). These results indicate that there are a large number of DEGs between the 227 and 228 inbred lines, and the number of DEGs decreases sequentially with increasing periods. The large number of DEGs between the 227 and 228 inbred lines may be related to differences in fatty acid synthesis.

### 2.6. The Functional Annotation of DEGs

The functions of DEGs between the 227 and 228 inbred lines have been annotated. GO enrichment analysis showed that a total of 2247 DEGs in the 227 and 228 inbred lines were enriched in GO function at the 10, 20, and 30 DAFB, with these DEGs enriched under 29 items. Among them, 9 items played a role in biological processes (BP); 18 items exercised molecular function (MF); and 2 items were involved in cellular component (CC) related activities. The Q_values_ (Q_value_ < 0.001) of GO entries at the 10, 20, and 30 DAFB were arranged in ascending order, with the top 10 GO items listed. The results showed that at the 10 DAFB, DEGs between 227 and 228 inbred lines mainly exhibited activities of omega-6 fatty acid desaturase, UDP-glucosyltransferase, quercetin 3-O-glucosyltransferase, quercetin 7-O-glucosyltransferase, glucosyltransferase, oxidoreductase, quercetin 4’-O-glucosyltransferase, gibberellin 20-oxidase, and their response to gibberellin (Table 1); At the 20 DAFB, DEGs between the 227 and 228 inbred lines mainly exhibited omega-6 fatty acid desaturase, UDP-glucosyltransferase, glucosyltransferase, microtubule motor, pigment binding, chlorophyll binding, tetrapyrrole binding, and light harvesting in photosystem I, as well as microtubule-associated complex components (Table 2); At the 30 DAFB, DEGs between the 227 and 228 inbred lines mainly exhibited activities of omega-6 fatty acid desaturase, UDP-glucosyltransferase, phosphatidylcholine 12-monooxygenase, glucosyltransferase, quercetin 3-O-glucosyltransferase, quercetin 7-O-glucosyltransferase, and AMP binding activity (Table 3). These results showed that the DEGs of the 227 and 228 inbred lines were mainly correlated with the activity of omega-6 fatty acid desaturase and glucosyltransferase.

### 2.7. The Screening and Analysis of DEGs

**Figure 5:**
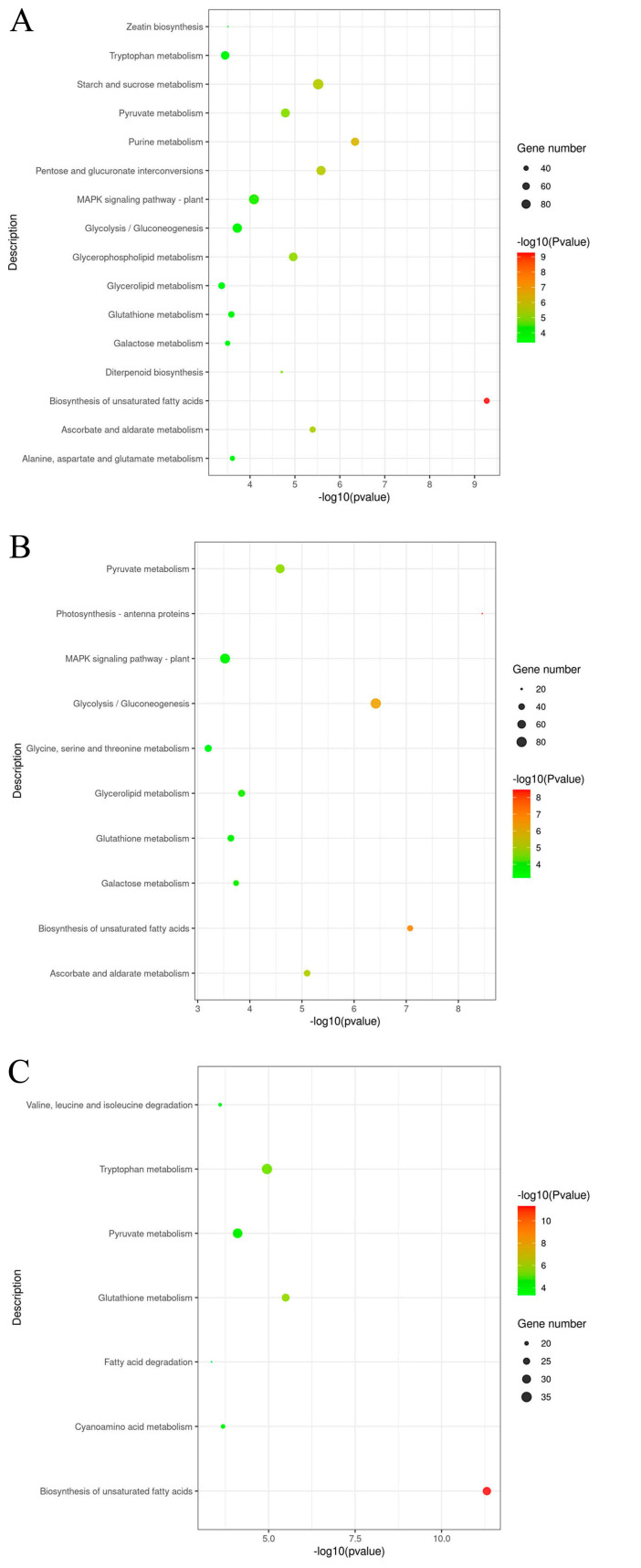
The KEGG pathways with significantly enriched DEGs between the 227 and 228 inbred lines. The *X*-axis represents the enrichment ratio, the *Y*-axis represents the KEGG enriched pathways, the size of bubbles represents the number of genes annotated onto a certain KEGG pathway, and the color represents the enrichment significance value Q-value. (**A**) At 10 DAFB, the KEGG pathway is significantly enriched in DEGs between the 227 and 228 inbred lines. (**B**) At 20 DAFB, the KEGG pathways are significantly enriched in DEGs between the 227 and 228 inbred lines. (**C**) At 30 DAFB, the KEGG pathways are significantly enriched in DEGs between the 227 and 228 inbred lines.

To further explore the biological functions of different gene products, KEGG was used to analyze the enriched pathways of DEGs. Using *p* value < 0.05 as the threshold, KEGG was used to analyze the enriched pathways of DEGs. The results showed that DEGs were significantly enriched in 16 metabolic pathways at 10 DAFB, including unsaturated fatty acid biosynthesis pathway, purine metabolism pathway, pentose and glucuronic acid interconversion pathway, starch and sucrose metabolism pathway, ascorbic acid and aldehyde ester metabolism pathway, glycerophospholipid metabolism pathway, pyruvic acid metabolism pathway, diterpene biosynthesis pathway, MAPK signaling pathway, glycolysis/glucose generation pathway, alanine, aspartate and glutamate metabolism pathway, glutathione metabolism pathway, galactose metabolism pathway, zeaxanthin synthesis pathway, tryptophan metabolism pathway, and glyceryl ester metabolism pathway. The DEGs of the 227 and 228 inbred lines at 20 DAFB were significantly enriched in 10 metabolic pathways, including the photosynthesis tentacle protein pathway, unsaturated fatty acid biosynthesis pathway, glycolysis/glucose generation pathway, ascorbic acid and aldehyde ester metabolism pathway, pyruvate metabolism pathway, glyceryl ester metabolism pathway, galactose metabolism pathway, glutathione metabolism pathway, MAPK signaling pathway, and glycine, serine, and threonine metabolism pathway. The DEGs of the 227 and 228 inbred lines at 30 DAFB were significantly enriched in seven metabolic pathways, namely unsaturated fatty acid synthesis pathway, glutathione metabolism pathway, tryptophan metabolism pathway, pyruvate metabolism pathway, cyanuric acid metabolism pathway, valine, leucine, and isoleucine degradation pathway, and fatty acid metabolism pathway (Tables S4–S6 and Figure 5). The results showed that there were significant differences in gene expression levels in the unsaturated fatty acid synthesis pathway, glutathione metabolism pathway, and pyruvate metabolism pathway between the high oleic acid inbred line 227 and the low oleic acid inbred line 228. These pathways may play an important role in influencing oleic acid synthesis.

### 2.8. The GO Mapping of DEGs Significantly Enriched Pathways

**Figure 6:**
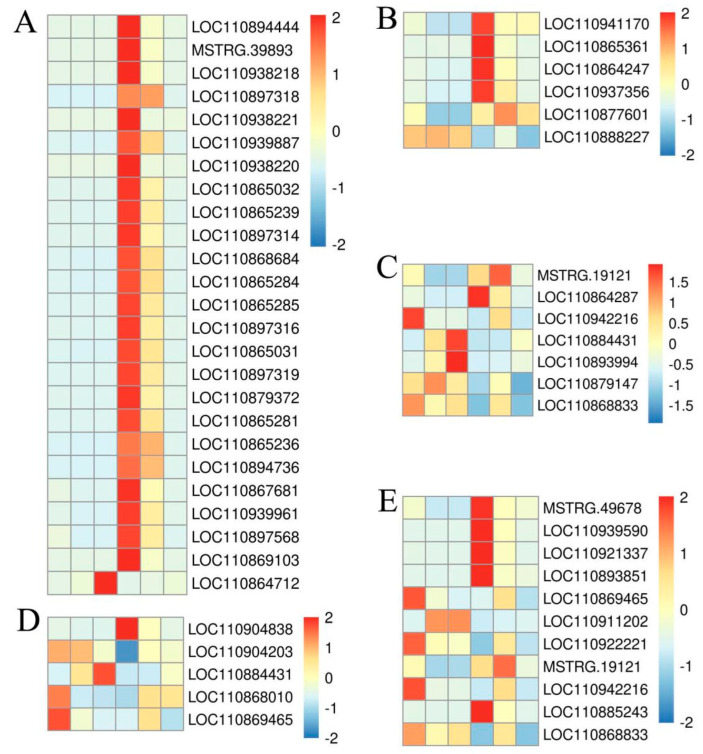
The expression levels of key genes enriched in the KEGG pathway. The horizontal axis represents the average gene expression levels of the 10, 20, and 30 DAFB of the 227 and 228 inbred lines from left to right, respectively. (**A**) The key genes are significantly enriched in the unsaturated fatty acid synthesis pathway (ko01040). (**B**) The key genes are significantly enriched in the glutathione metabolism pathway (ko00480). (**C**) The key genes are significantly enriched in the pyruvate metabolism pathway (ko00620). (**D**) The key genes are significantly enriched in the glycerol ester metabolism pathway (ko00561). (**E**) The key genes are significantly enriched in the glycerophospholipid metabolism pathway (ko00564).

Among the DEGs enriched in GO and KEGG pathways, genes with FPKM > 3 between the 227 and 228 inbred lines were screened. We analyzed the key genes of the KEGG pathway related to controlling oleic acid content. We found that the DEGs were significantly enriched in the unsaturated fatty acid synthesis pathway (ko01040), glutathione metabolism pathway (ko00480), and pyruvate metabolism pathway (ko00620) (Figure 6). Through analyzing the unsaturated fatty acid synthesis pathway, a total of 23 omega-6 fatty acid desaturase genes (*FAD2/FAD6*) were significantly upregulated at 10 DAFB (LOC110894444, MSTRG.39893, LOC110938218, LOC110897318, LOC110938221, LOC110939887, LOC110938220, LOC110865032, LOC110865239, LOC110897314, LOC110868684, LOC110865284, LOC110865285, LOC110897316, LOC110865031, LOC110897319, LOC110879372, LOC110865281, LOC110865236, LOC110894736, LOC110894736, LOC110867681, LOC110110939961 and LOC110897568), 3-oxo-5-α-steroido-4-dehydrogenase gene (LOC110869103) was upregulated. The palmitoyl monoacyl β-7 desaturase gene (LOC110864712) of galactosyl diacylglycerol was downregulated (Figure 6A). The acid desaturase gene (LOC110904312) was downregulated at 10 DAFB and upregulated at 20 and 30 DAFB. In the glutathione metabolic pathway, the methyltransferase-like protein 17 gene (LOC110941170), glutathione transferase genes (LOC110865361, LOC110864247, and LOC110937356), and leucine aminopeptidase gene (LOC110877601) were upregulated; The glutathione S-transferase gene DHAR3 (LOC110888227) was downregulated in expression (Figure 6B). In the pathway of pyruvic acid metabolism, the dihydrothioyl dehydrogenase gene (LOC110891536), ethanol dehydrogenase gene (MSTRG. 19121), and L-lactic acid dehydrogenase B gene (LOC110864287) were upregulated, while the ethanol dehydrogenase gene (LOC110942216), aldehyde reductase gene (LOC110884431), malate enzyme gene (LOC110893994), NADP dependent D-sorbitol 6-phosphate dehydrogenase protein gene (LOC110879147), and zinc binding dehydrogenase gene (LOC110868833) were downregulated (Figure 6C). In addition, during the 10 and 20 DAFB, DEGs were significantly enriched in the glycerol ester metabolism pathway (ko00561) and glycerophospholipid metabolism pathway (ko00564). In the glycerol ester metabolism pathway, the glycerol-3-phosphate acyltransferase genes (LOC110899775 and LOC110904838) were upregulated, while the zinc binding dehydrogenase gene (LOC110922221), diacylglycerol acyltransferase 2 gene (LOC110904203), aldose reductase (LOC110884431), glutaredoxin gene (LOC110868010), and diacylglycerol kinase gene (LOC110869465) were downregulated. In the glycerophospholipid metabolism pathway, phospholipase genes (LOC110894725), ribonuclease H protein genes (MSTRG. 49678), phosphatidylcytidine transferase 1 genes (LOC110939590), glycerophosphodiesterase genes (LOC110921337), glycerol-3-phosphate acyltransferase genes (LOC110904838 and LOC110899775), and phospholipase D genes (LOC110893851) were upregulated, while diacylglycerol kinase genes (LOC110869465), glycerodiphosphate phosphodiesterase genes (LOC110911202), and lipid phosphatase genes (LOC110922221) were downregulated (Figure 6D,E). At 30 DAFB, DEGs were significantly enriched in the fatty acid metabolism pathway (ko00071). The ethanol dehydrogenase gene ADH1 (MSTRG. 19121) was upregulated, while LOC110942216 was downregulated; the long-chain acyl-CoA synthase gene (LOC110885243) was upregulated; the zinc binding dehydrogenase gene (LOC110868833) was downregulated (Figure 6C,E).

### 2.9. Co-Expression Network Analysis

**Figure 7:**
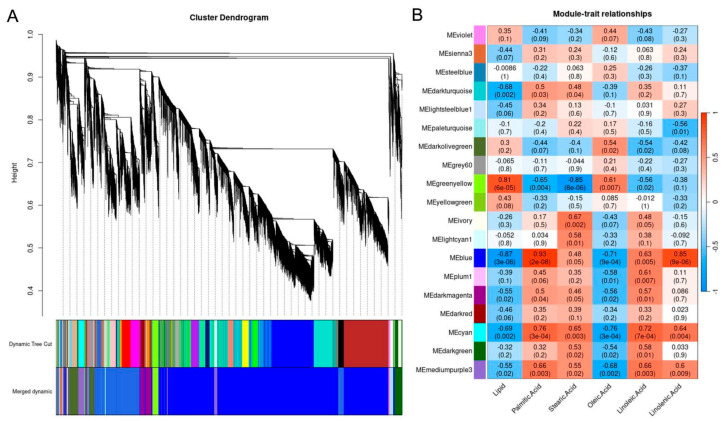
(**A**) The gene module detection of WGCNA. (**B**) Correlation between WGCNA co-expression gene module and phenotype.

All DEGs were used for WGCNA co-expression network analysis. The results showed the DEGs were enriched in 19 modules (Figure 7). The soft threshold power of 6 (β = 6) was selected according to the preconditions of approximate scale-free topology (Figure S2). The correlation coefficients of DEGs were high in the MEgreenyellow and MEblue modules (Figure 7B), indicating a consistent expression pattern. There were a total of 18,231 DEGs on the MEblue module, but they were not significantly enriched in the KEGG pathway. On the MEblue module, GO was significantly enriched in 30 entries, with entry GO:0045485 containing 16 omega-6 fatty acid desaturase genes. Among them, 15 genes—LOC110867681, LOC110865032, LOC110879372, LOC110897319, LOC110865285, LOC110865239, LOC110897314, LOC110865031, LOC110897316, LOC110868684, LOC110939961, LOC110939887, LOC110897568, LOC110894736, and LOC110897318—are consistent with the enriched genes in the GO mapping of 3.7 the GO Mapping of DEGs significantly enriched pathways. There were a total of 568 genes on the MEgreenyellow module, mainly enriched in pyruvate metabolism, glutathione metabolism, protein processing in the endoplasmic reticulum, and carbon fixation in photosynthetic tissue. GO was significantly enriched in 19 entries, but was not enriched in genes consistent with 3.7 the GO Mapping of significantly enriched DEGs pathways (Figure 7).

### 2.10. qRT-PCR Validation

**Figure 8:**
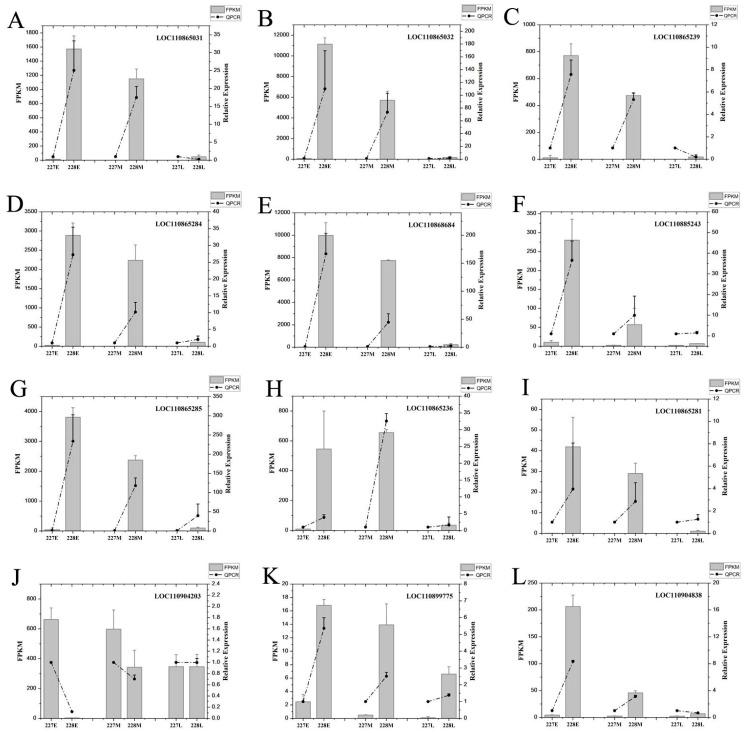
The validation of DEGs from RNA-seq using qRT-PCR with 18S rRNA as an internal reference. (**A**) LOC110865031, (**B**) LOC110865032, (**C**) LOC110865239, (**D**) LOC110865284, (**E**) LOC110868684, (**F**) LOC110885243, (**G**) LOC110865285, (**H**) LOC110865236, (**I**) LOC110865281, (**J**) LOC110904203, (**K**) LOC110899775 and (**L**) LOC110904838 are the validation of DEGs of different genes in RNA seq. The gray bars represent the average FPKM values (SD, error bars) of the 227 and the 228 inbred lines at 10, 20, and 30 DAFB. The dashed line connecting the gray bars represents the average of the three biological replicates of qRT-PCR.

To verify the accuracy of the transcriptome analysis, 16 genes related to oleic acid metabolism were selected for RT-qPCR analysis. 18S rRNA was used as an internal reference Table S7. The results showed that the FPKM and relative expression of all 12 genes were consistent at 10 and 20 DAFB. At 30 DAFB, three genes were inconsistent (LOC110865031, LOC110865239, and LOC11094838), which might be due to the fact that the gene expression levels were all relatively low. The overall concordance rate was 83%, indicating the reliability of the transcriptome results (Figure 8).

## 3. Discussion

Sunflower oil containing high monounsaturated fatty acids (oleic acid) has higher antioxidant stability during cooking, processing, or storage [2,15]. Improving the oleic acid content of sunflower seeds is an important goal of genetic improvement in sunflowers [2]. In this study, high oleic acid inbred line 227 and low oleic acid inbred line 228 were used as materials to identify genes affecting sunflower oleic acid synthesis through transcriptome analysis. We found that DEGs between 227 and 228 inbred lines were significantly enriched in the unsaturated fatty acid synthesis pathway, glutathione metabolism pathway, and pyruvate metabolism pathway at 10, 20, and 30 DAFB. At 10 and 20 DAFB, significant enrichment was observed in the glycerol ester metabolism pathway and glycerophospholipid metabolism pathway. Previous researchers have discovered multiple pathways that affect fatty acid synthesis. The regulation of carbon source allocation alters the composition of fatty acids. The increased activity of pyruvate carboxylase (PEPCase) in the pyruvate metabolism pathway promotes the conversion of more carbon sources to proteins and inhibits the conversion of carbon sources to lipid synthesis [16]. The silencing of the *GhPEPC1* gene in cotton leads to an increase in the expression level of oil synthesis-related genes, ultimately resulting in a 16.7% increase in oil content in transgenic cottonseeds [17]. Changing the activity of key enzymes involved in fatty acid synthesis can significantly alter oil content and fatty acid composition. Overexpression of the acetyl-CoA carboxylase (ACCase) gene in the fatty acid synthesis pathway significantly increases the oil content of upland cotton seeds [18]. Overexpression of fatty acid synthase genes *GhKAR* and *GhENR* in upland cotton effectively increased the oil content of cottonseeds, and the relative content of unsaturated fatty acids in cottonseeds increased by about 10% compared to control plants [19]. In Arabidopsis, the decrease in KAS II expression level increased the proportion of stearic acid and decreased the proportion of oleic acid [20]. In shepherd’s purse, knocking out the *FATB* gene reduced palmitic acid content by 45%, stearic acid content by 38%, and total saturated fatty acid content by 35% [21]. Changes in key enzyme genes involved in the assembly pathway of triglycerides can regulate fatty acid composition. Overexpression of *AtGPAT*, *AtDGAT*, and *AtPDAT* genes in the biosynthesis pathway of triglycerides in Arabidopsis increased seed oil content. Inhibition of *AtGPAT9* gene expression leads to a decrease in oil content in Arabidopsis seeds [22]. Overexpression of *AtDGAT1* in Arabidopsis seeds significantly increased DGAT activity by 10–70%, leading to an increase in seed oil content [23]. Our research is consistent with previous studies, further demonstrating that the fatty acid synthesis pathway, pyruvate metabolism pathway, and triglyceride synthesis pathway play important roles in affecting plant oleic acid synthesis, providing key pathways and genes for the breeding of high oleic sunflower varieties.

The ω-6 fatty acid dehydrogenases (ω-6 FADs) are located in the binding membranes of endoplasmic reticulum membranes, plastid membranes, and other parts of plant cells. They can alter the composition of unsaturated fatty acids in plants, and their key role is to insert oleic acid into the double bond to form linoleic acid [14,24] (Figure 6). The coding sequences of ω-6 fatty acid desaturase in plants can be divided into three categories: housekeeping type FAD2, and seed types FAD2 and FAD6 [25,26]. The ω-fatty acid desaturases FAD2 and FAD6 can both catalyze the desaturation reaction of oleic acid to produce linoleic acid [27,28,29]. Overexpression of *FAD2-5* and *FAD2-2* genes in olives plays a crucial role in the production of oleic acid in their skin [30]. The deletion mutants of the *BnFAD2-1* and *BnFAD2-2* genes at the E106K and G303E loci in rapeseed exhibit high oleic acid characteristics [31]. *FAD2* gene editing in *Brassica napus* increased the oleic acid content in seeds [32]. Most studies related to high oleic acid crops have focused on preventing or reducing the flux of 18:1 to 18:2 controlled by the FAD2 family [33,34,35]. In this study, we identified differential expression of 23 omega-6 fatty acid desaturase genes FAD2/FAD6. The FAD2/FAD6 genes were significantly upregulated, promoting the conversion of oleic acid to linoleic acid, resulting in a lower oleic acid content in the low oleic acid inbred line 228 compared to the 227 inbred line.

Diacylglycerol acyltransferase (DGAT) is the rate-limiting enzyme for triacylglycerol synthesis (Figure 6), and its expression affects plant seed development, oil content, and fatty acid composition [14,36]. Overexpression of *AtDGAT1* resulted in a 10% to 70% increase in DGAT activity in transgenic Arabidopsis seeds compared to the wild type, as well as higher thousand-grain weight and oil content [23]. *DGAT2* is considered to be the main controlling gene for the accumulation of specific fatty acids, and it is also involved in the accumulation of triglycerides in olives [37] and oil palm [38]. Expression of short-chain *S-NcDGAT2* from the fungus Aspergillus oryzae in maize increased oleic acid content and decreased linoleic acid content [39]. In this study, we identified a downregulated expression of a diacylglycerol acyltransferase 2 gene (LOC110904203). Based on previous research, we speculate that the downregulation of diacylglycerol acyltransferase 2 gene expression may be related to the decrease in oleic acid content in the low oleic acid sunflower inbred line 228.

Glycerol-3-phosphate is esterified to triacylglycerol (Kennedy pathway) through glycerol-3-phosphate acyltransferase (GPAT), lysophosphatidoyl transferase (LPAAT), and diacylglyceroyl transferase (DGAT), respectively [14,22,40,41]. The absence of *GPAT1* in Arabidopsis results in a decrease in saturated fatty acid content and an increase in polyunsaturated fatty acid content [42]. In camelina, the *Csdgat1* homozygous mutant showed an increase in 18:2 content and a decrease in 18:3 content. The *Cspdat1* homozygous mutant exhibited a fatty acid composition similar to the wild type, but with a decrease in oil content [43]. In this study, we found that glycerol-3-phosphate acyltransferase genes (LOC110899775 and LOC110904838) were upregulated. Based on previous research, we speculate that the decrease in oleic acid content in the 228 inbred line may be related to the upregulation of glycerol-3-phosphate acyltransferase gene expression.

Long-chain acyl-CoA synthetase (LACS) catalyzes the conversion of fatty acids to acyl-CoA and plays an important role in plant lipid metabolism (Figure 6) [14]. Although there is some understanding of the functions of *LACSs* in Arabidopsis and soybean, the understanding of their roles in common crops is still very limited [44]. Overexpression of the *GmACSL2* gene in yeast and soybean hairy roots significantly reduced lipid content [45]. In this study, we found that a long-chain acyl-CoA synthase gene (LOC110885243) was upregulated. Previous studies have found that *LACS* affects plant lipid content, but whether it affects oleic acid/linoleic acid composition has not been reported, and further research is needed.

## 4. Materials and Methods

### 4.1. Sunflower Plant Materials

High oleic sunflower inbred line 227 and low oleic sunflower inbred line 228 were used as experimental materials. The 227 and 228 inbred lines were planted in the experimental field of Inner Mongolia Academy of Agricultural and Animal Husbandry Sciences in Hohhot on 14 May 2023, with each plot covering an area of 21 square meters. Three replicates were set up, and the same cultivation and agricultural practices were used for the experimental materials.

### 4.2. Fatty Acid Composition and Content Determination

Sunflower seeds from 10 days (S1 stage), 20 days (S2 stage), and 30 days (S3 stage) after pollination were taken as experimental samples for the determination of fatty acid content. The content of oleic acid, linoleic acid, stearic acid, linolenic acid, and palmitic acid in each sample was determined using liquid chromatography.

### 4.3. Sample Collection

The sunflower seeds from 10, 20, and 30 DAFB were used as experimental samples for RNA-seq. Each sample was repeated 3 times, and a total of 18 samples were collected and frozen in liquid nitrogen.

### 4.4. Determination of Fats and Fatty Acids

According to the national standard of the People’s Republic of China “Determination of Fat in Foods” GB5009.6-2016 [46]. The content of fatty acids was determined using the SZF-06A fat analyzer (Shanghai, China, Xinrui) [27]. In addition, the ratio of each fatty acid, oleic acid, linoleic acid, linolenic acid, and palmitic acid, was determined in the samples using the national standard of the People’s Republic of China (Determination of Fat in Food, GB5009.168-2016) [38,47].

### 4.5. RNA-Seq

Total RNA of sunflower seed samples was prepared using the NEBNext Ultra RNA Library Preparation Kit (NEB, USA, Catalog #: E7530L). The transcriptome sequencing was performed using an Illumina high-throughput sequencer. The mRNA was enriched with Oligo (dT) magnetic beads and then cut into short fragments (raw reads). The impure reads by the adapter, reads greater than 5% N, and low-quality reads were deleted, resulting in clean reads. The clean reads were spliced and aligned with the sunflower reference genome (GeneBank NO. GCA_002127325.2). The FPKM (kilobase fragment count per million mapped reads per transcript) value was calculated and used to estimate the impact of sequencing depth and gene length on read counts.

Differentially expressed genes (DEGs) analysis was performed using the DESeq2 R package (1.40.2). The threshold for DEGs was set to Padj ≤ 0.01 and |log_2_FoldChange| ≥ 1. GO and KEGG enrichment analysis was performed using the ClusterProfiler R package (4.8.3). Weighted co-expression network analysis was performed using the WGCNA R package (1.72-1).

### 4.6. RT-qPCR

To verify the accuracy of the transcriptome, 12 DEGs related to the function of oleic acid metabolism were selected for RT-qPCR experiments. The RT-qPCR analysis was performed using the Bio-Rad CFX96 (Bio-rad, Hercules, CA, USA) instrument. The primer sequences were designed by BioWorks Online and were listed in Table S1. 18S RNA was used as an internal reference, and relative expression was calculated using the 2^−△△CT^ method. The experiment was performed on the KK platform, and three technical replicates were set up for each sample.

## 5. Conclusions

This study aimed to explore the key functional genes that regulate the oleic acid content in sunflowers and further revealed the molecular mechanism and regulatory rules of sunflower oleic acid formation. RNA-seq was used to analyze differentially expressed genes between the high oleic acid strain 227 and the low oleic acid strain 228 of sunflowers at 10 days (S1), 20 days (S2), and 35 days (S3) after pollination. We found that in stages S1, S2, and S3, DEGs were significantly enriched in the unsaturated fatty acid synthesis pathway, glutathione metabolism pathway, and pyruvate metabolism pathway. In stages S1 and S2, DEGs were significantly enriched in the glycerolipid metabolism pathway and glycerophospholipid metabolism pathway; In the S3 phase, DEGs were significantly enriched in the fatty acid metabolism pathway. The comprehensive analysis of RNA-seq and qRT-PCR revealed that the omega-6 fatty acid desaturase genes (LOC110865032, LOC110865284, LOC110865285, LOC110865236, and LOC110865281), the long-chain acyl-CoA synthase gene (LOC110885243), the diacylglyceroyl transferase gene (LOC110904203), and the glycerol-3-phosphate acyltransferase gene (LOC110899775) may be important genes controlling the oleic acid content of sunflower seeds.

## Figures and Tables

**Table 1:** GO enrichment items of DEGs of 227 and 228 inbred lines at the 10 DAFB.

ID	Ontology	Description	Count	*p*-Value	Q-Value
GO:0045485	MF	omega-6 fatty acid desaturase activity	18	9.66 × 10^−11^	8.67 × 10^−8^
GO:0035251	MF	UDP-glucosyltransferase activity	106	3.38 × 10^−10^	1.51 × 10^−7^
GO:0080043	MF	quercetin 3-O-glucosyltransferase activity	75	7.74 × 10^−10^	1.74 × 10^−7^
mGO:0080044	MF	quercetin 7-O-glucosyltransferase activity	75	7.74 × 10^−10^	1.74 × 10^−7^
GO:0046527	MF	glucosyltransferase activity	126	5.69 × 10^−9^	1.02 × 10^−6^
GO:0008194	MF	UDP-glycosyltransferase activity	131	5.46 × 10^−8^	8.17 × 10^−6^
GO:0016705	MF	oxidoreductase activity, acting on paired donors, with incorporation or reduction of molecular oxygen	137	2.18 × 10^−7^	2.79 × 10^−5^
GO:0009739	BP	response to gibberellin	110	1.59 × 10^−8^	5.59 × 10^−5^
GO:0080046	MF	quercetin 4’-O-glucosyltransferase activity	22	1.73 × 10^−5^	0.001939238
GO:0045544	MF	gibberellin 20-oxidase activity	11	2.26 × 10^−5^	0.002252498

**Table 2:** GO enrichment items of DEGs of 227 and 228 inbred lines at the 20 DAFB.

ID	Ontology	Description	Count	*p*-Value	Q-Value
GO:0045485	MF	omega-6 fatty acid desaturase activity	16	5.98 × 10^−10^	5.33 × 10^−7^
GO:0005875	CC	microtubule-associated complex	58	1.68 × 10^−7^	7.72 × 10^−5^
GO:0031409	MF	pigment binding	14	5.97 × 10^−7^	0.000266049
GO:0009768	BP	photosynthesis, light harvesting in photosystem I	15	1.35 × 10^−7^	0.000464913
GO:0008194	MF	UDP-glycosyltransferase activity	102	2.61 × 10^−6^	0.00046543
GO:0016168	MF	chlorophyll binding	14	2.42 × 10^−6^	0.00046543
GO:0035251	MF	UDP-glucosyltransferase activity	78	1.71 × 10^−6^	0.00046543
GO:0046527	MF	glucosyltransferase activity	95	3.33 × 10^−6^	0.000494834
GO:0046906	MF	tetrapyrrole binding	24	1.13 × 10^−5^	0.001435517
GO:0003777	MF	microtubule motor activity	42	2.45 × 10^−5^	0.002235062

**Table 3:** GO enrichment items of DEGs of 227 and 228 inbred lines at the 30 DAFB.

ID	Ontology	Description	Count	*p*-Value	Q-Value
GO:0045485	MF	omega-6 fatty acid desaturase activity ω-6	16	3.50 × 10^−16^	2.69 × 10^−13^
GO:0035251	MF	UDP-glucosyltransferase activity	45	2.77 × 10^−8^	1.06 × 10^−5^
GO:0050183	MF	phosphatidylcholine 12-monooxygenase activity	8	1.52 × 10^−7^	3.90 × 10^−5^
GO:0016208	MF	AMP binding	10	3.03 × 10^−7^	5.01 × 10^−5^
GO:0046527	MF	glucosyltransferase activity	51	3.26 × 10^−7^	5.01 × 10^−5^
GO:0008194	MF	UDP-glycosyltransferase activity	53	7.86 × 10^−7^	0.000100833
GO:0080043	MF	quercetin 3-O-glucosyltransferase activity	29	6.59 × 10^−6^	0.000633808
GO:0080044	MF	quercetin 7-O-glucosyltransferase activity	29	6.59 × 10^−6^	0.000633808

## Data Availability

The authors do not have permission to share data.

## Supplementary Materials

The following supporting information can be downloaded at: https://www.mdpi.com/article/10.3390/ijms26146757/s1.
