# Supplementary material for: Transcriptome Analysis Reveals Metabolic Pathways and Key Genes Involved in Oleic Acid Formation of Sunflower (Helianthus annuus L.)
Source: Int J Mol Sci. 2025 Jul 15;26(14):6757. doi: 10.3390/ijms26146757 (PMC12295131; doi:10.3390/ijms26146757)
Supplement: Supplementary file 1 [file ijms-26-06757-s001.zip › ijms-3607636-supplementary.pdf]

Supplementary Table S1. The primer sequences used in the amplification in the RT-qPCR.

Gene	Forward primer	Reverse primer
LOC110865031	TACAGCCACCGTAGCCACCAC	GTGTCGTTCTTGCGTTTGGGAATG
LOC110865032	CGCCACTTGTTATGCCGTTTATCG	GAGGGATAGCAGGGTGGGTATGG
LOC110865236	ATCGGAACCACCATGCCAACAC	GTGAACACTCGACCTGGAGGATTG
LOC110865239	TGTGTCTATGGTGGTGCGTTGATG	AACGAAGGGTGCGTATGATGAAGG
LOC110865281	ATGACGCTGTTGGGTTTGTCCTC	TTCGTGTTGGCATGGTGGTTCC
LOC110865284	TTATCGGTCACGAATGCGGTCAC	AGAGCAGAGTGGAGGACGAACC
LOC110865285	TCATCGTCGTCACCATTCCAACAC	AGCACAAGACCACAAGCCTGAAG
LOC110868684	CCACCATGCCAACACCAACTCC	GGGAAGCCGAGAACGATCTTGAAC
LOC110885243	GCGTCTCCGTTGTCTGCTGATG	GTGACCTGAGGTGACATCGTTGAC
LOC110868937	TCCACTTCATCCACGCCAATGC	CGCAAGCAACGGGTTGAAATGAC
LOC110941520	TGGTGGTTGTCTGGTTAAGTTGGC	TGTTTGAACGCATTAGGGGTTGGG
LOC110903993	CCGCTCGTTGACCTTGCTGAC	GGAGTTGTGGAAACCCGTGACC
18S	CATCCGATGACTTCTTTCGGAA	CCAGTCATCATCTTCTTGCTGA

Supplementary Table S2. The quality analysis of sequencing samples.

Sample	Raw Reads	Clean Reads	Raw Base(G)	Clean Base(G)	Effective (%)	Error(%)	Q20(%)	Q30(%)	GC(%)
227s1.1	42244490	41386968	6.34	6.21	97.97	0.03	97.84	94.08	46.18
227s1.2	41612494	40958212	6.24	6.14	98.43	0.03	97.79	93.9	44.87
227s1.3	41145644	40481228	6.17	6.07	98.39	0.02	98.09	94.6	46.04
227s2.1	41791290	41387760	6.27	6.21	99.03	0.02	98.1	94.68	47.73
227s2.2	41785976	41029530	6.27	6.15	98.19	0.02	98.11	94.66	47.24
227s2.3	42365966	415 68474	6.35	6.24	98.12	0.03	97.86	94.04	47.37
227s3.1	42602930	41509720	6.39	6.23	97.43	0.02	97.99	94.36	47.62
227s3.2	41332002	40389764	6.2	6.06	97.72	0.02	98.1	94.67	47.68
227s3.3	43116788	42203900	6.47	6.33	97.88	0.02	98.06	94.6	47.85
228s1.1	40789028	40155576	6.12	6.02	98.45	0.03	97.66	93.58	43.84
228s1.2	41936900	41403008	6.29	6.21	98.73	0.02	98.03	94.45	44.09
228s1.3	41568146	40856682	6.24	6.13	98.29	0.02	98.08	94.52	44.05
228s2.1	42165230	41430504	6.32	6.21	98.26	0.02	98.02	94.49	45.65
228s2.2	44091944	43052420	6.61	6.46	97.64	0.03	97.41	92.98	45.65
228s2.3	42170202	41395866	6.33	6.21	98.16	0.02	97.97	94.25	45.13
228s3.1	43510650	42670324	6.53	6.4	98.07	0.02	97.99	94.38	48.2
228s3.2	41402050	40357214	6.21	6.05	97.48	0.02	97.92	94.3	47.15
228s3.3	39386122	38406986	5.91	5.76	97.51	0.03	97.89	94.16	47.66

Supplementary Table S3. Differences of gene expression levels between the 227 and 228 inbred lines.

Inbred lines	227 inbred line			228 inbred line		
Stages	S1	S2	S3	S1	S2	S3
FPKM＜1	74089	79431	80572	73677	73084	78368
1＜FPKM＜10	19013	17794	16737	17532	19247	18254
FPKM＞10	9013	4890	4806	10906	9784	5493

Supplementary Table S4. The KEGG enrichment pathways of DEGs between strain 227 and 228 at S1 stage

ID	Description	Count	pvalue	qvalue
ko01040	Biosynthesis of unsaturated fatty acids	45	5.37E-10	1.47E-07
ko00230	Purine metabolism	67	4.58E-07	3.15E-05
ko00040	Pentose and glucuronate interconversions	81	2.62E-06	0.000138957
ko00500	Starch and sucrose metabolism	98	3.03E-06	0.000138957

ko00053	Ascorbate and aldarate metabolism	47	4.03E-06	0.000158042
ko00564	Glycerophospholipid metabolism	73	1.09E-05	0.000299424
ko00620	Pyruvate metabolism	76	1.63E-05	0.000375866
ko00904	Diterpenoid biosynthesis	28	1.97E-05	0.000385925
ko04016	MAPK signaling pathway - plant	93	8.16E-05	0.001401171
ko00010	Glycolysis / Gluconeogenesis	83	0.00019192	0.002775131
ko00250	Alanine, aspartate and glutamate metabolism	39	0.000246054	0.003380006
ko00480	Glutathione metabolism	49	0.000259788	0.003398731
ko00052	Galactose metabolism	40	0.000315258	0.00376578
ko00908	Zeatin biosynthesis	27	0.00031026	0.00376578
ko00380	Tryptophan metabolism	70	0.00035753	0.003984208
ko00561	Glycerolipid metabolism	51	0.000426814	0.004510061

Supplementary Table S5. The KEGG enrichment pathways of DEGs between strain 227 and 228 at S2 stage

ID	Description	Count	pvalue	qvalue
ko00196	Photosynthesis - antenna proteins	19	3.48E-09	2.13E-07
ko01040	Biosynthesis of unsaturated fatty acids	36	8.42E-08	2.95E-06
ko00010	Glycolysis / Gluconeogenesis	80	3.84E-07	1.18E-05
ko00053	Ascorbate and aldarate metabolism	40	7.97E-06	0.000195399
ko00620	Pyruvate metabolism	64	2.62E-05	0.000583309
ko00561	Glycerolipid metabolism	45	0.000143908	0.002076195
ko00052	Galactose metabolism	35	0.000183322	0.002366427
ko00480	Glutathione metabolism	42	0.00023102	0.002698125
ko04016	MAPK signaling pathway - plant	76	0.000297965	0.003321805
ko00260	Glycine, serine and threonine metabolism	43	0.000626792	0.006149156

Supplementary Table S6. The KEGG enrichment pathways of DEGs between strain 227 and 228 at S3 stage

ID	Description	Count	pvalue	qvalue
ko01040	Biosynthesis of unsaturated fatty acids	28	4.90E-12	1.41E-09
ko00480	Glutathione metabolism	27	3.22E-06	0.000154019
ko00380	Tryptophan metabolism	35	1.11E-05	0.0004575
ko00620	Pyruvate metabolism	33	7.87E-05	0.00188507
ko00460	Cyanoamino acid metabolism	20	0.000207702	0.0045913
ko00280	Valine, leucine and isoleucine degradation	19	0.000252013	0.005172901
ko00071	Fatty acid degradation	18	0.000446241	0.008549033

Supplementary Table S7. The specific primer sequences of qRT-PCR-related genes

Gene ID	Primer sequence (5'-3')
LOC110865031	TACAGCCACCGTAGCCACCAC<br>GTGTCGTTCTTGCGTTTGGGAATG
LOC110865032	CGCCACTTGTTATGCCGTTTATCG<br>GAGGGATAGCAGGGTGGGTATGG
LOC110865236	ATCGGAACCACCATGCCAACAC

	GTGAACACTCGACCTGGAGGATTG
LOC110865239	TGTGTCTATGGTGGTGCGTTGATG<br>AACGAAGGGTGCGTATGATGAAGG
LOC110865281	ATGACGCTGTTGGGTTTGTCCTC<br>TTCGTGTTGGCATGGTGGTTCC
LOC110865284	TTATCGGTCACGAATGCGGTCAC<br>AGAGCAGAGTGGAGGACGAACC
LOC110865285	TCATCGTCGTCACCATTCCAACAC<br>AGCACAAGACCACAAGCCTGAAG
LOC110868684	CCACCATGCCAACACCAACTCC<br>GGGAAGCCGAGAACGATCTTGAAC
LOC110879372	TTATCCCGCGCCTCCCTTATCC<br>CCAAATGCCCATGAACACACAACC
LOC110885243	GCGTCTCCGTTGTCTGCTGATG<br>GTGACCTGAGGTGACATCGTTGAC
LOC110868937	TCCACTTCATCCACGCCAATGC<br>CGCAAGCAACGGGTTGAAATGAC
LOC110941520	TGGTGGTTGTCTGGTTAAGTTGGC<br>TGTTTGAACGCATTAGGGGTTGGG
LOC110903993	CCGCTCGTTGACCTTGCTGAC<br>GGAGTTGTGGAAACCCGTGACC
LOC110904203	TGTCGCATGGCATAGCAACTGATC<br>CCGGAGCATCTGGCAACTTGG
LOC110899775	CAAGTGGCGGCACGGACATG<br>TGAAGCAGTTCTGAAGCGGATCTC
LOC110904838	ATGGTTCCGTGGGATGGATTCTTG<br>TTGTCTTCCAGACGACGCAACAC
18S	CATCCGATGACTTCTTTCGGAA<br>CCAGTCATCATCTTCTTGCTGA

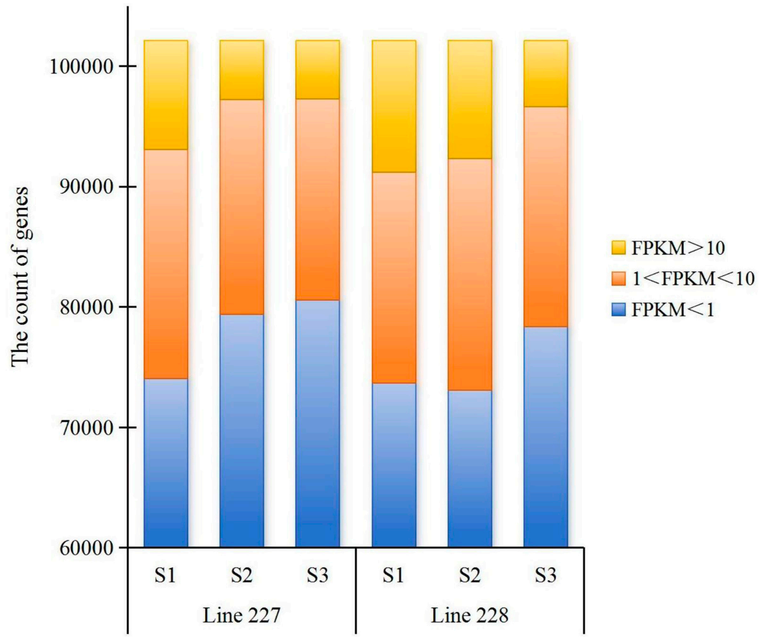

Supplementary Figure S1. The histogram of gene expression levels of the samples.

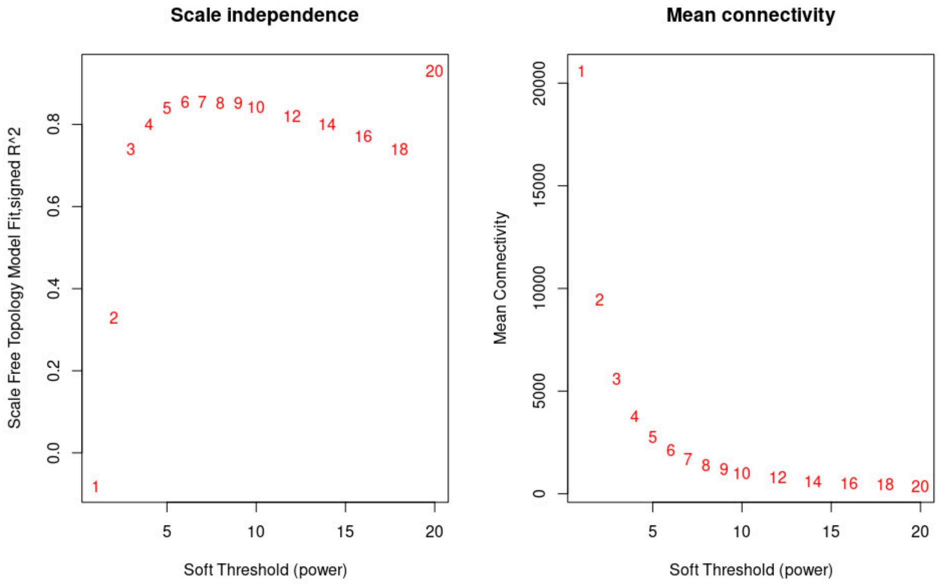

Supplementary Figure S2. The threshold analysis of WGCNA
